# Perinatal Depression and the Role of Synaptic Plasticity in Its Pathogenesis and Treatment

**DOI:** 10.3390/bs13110942

**Published:** 2023-11-17

**Authors:** Sonia Shenoy, Sufyan Ibrahim

**Affiliations:** 1 Department of Psychiatry, Kasturba Medical College, Manipal, Manipal Academy of Higher Education, Manipal 576104, Karnataka, India; sonia.shenoy@manipal.edu; 2 Neuro-Informatics Laboratory, Mayo Clinic, Rochester, MN 55902, USA

**Keywords:** synaptic plasticity, perinatal depression, depression, neuromodulation

## Abstract

Emerging evidence indicates that synaptic plasticity is significantly involved in the pathophysiology and treatment of perinatal depression. Animal models have demonstrated the effects of overstimulated or weakened synapses in various circuits of the brain in causing affective disturbances. GABAergic theory of depression, stress, and the neuroplasticity model of depression indicate the role of synaptic plasticity in the pathogenesis of depression. Multiple factors related to perinatal depression like hormonal shifts, newer antidepressants, mood stabilizers, monoamine systems, biomarkers, neurotrophins, cytokines, psychotherapy and electroconvulsive therapy have demonstrated direct and indirect effects on synaptic plasticity. In this review, we discuss and summarize the various patho-physiology-related effects of synaptic plasticity in depression. We also discuss the association of treatment-related aspects related to psychotropics, electroconvulsive therapy, neuromodulation, psychotherapy, physical exercise and yoga with synaptic plasticity in perinatal depression. Future insights into newer methods of treatment directed towards the modulation of neuroplasticity for perinatal depression will be discussed.

## 1. Introduction

Synaptic plasticity is a fascinating ability of the human brain to modify the neural circuit behavior and in turn influence future thoughts, emotions, and behaviors. Synaptic plasticity is defined as the enhancement or reduction of synaptic transmission at pre-existing synapses which can range from a few milliseconds (short-term) to days and even longer duration (long-term) [1]. Short-term synaptic plasticity is noted at literally all synapses in almost all organisms and has a role in short-term adaptations to sensory stimuli, short-lasting memory, and transient behavioral modifications. Short-term synaptic plasticity is usually initiated by short-lasting bursts of activity that cause temporary accumulation of calcium in presynaptic nerve terminals, which in turn induces modifications in the release of neurotransmitters by directly altering the biochemical processes underlying the exocytosis of synaptic vesicles. Long-term synaptic plasticity is noted after repetitive stimulation of synapses with longer-lasting (usually 200 milliseconds to 5 s) trains of stimulation applied at higher frequencies such as 10–200 Hz [2]. Long-term synaptic plasticity has a key role in learning and memory as it can bi-directionally alter synaptic strength by either enhancing (long-term potentiation or LTP) or depressing (long-term depression or LTD) it. Post-synaptic plasticity usually includes alterations in the number or properties of the postsynaptic receptors, whereas pre-synaptic plasticity includes changes in the amount of release of neurotransmitters [3]. Synaptic plasticity is known to play a role in the pathogenesis of numerous neuropsychiatric disorders, including depression.

Here, we attempt to discuss a broad overview of the role of synaptic plasticity in the pathogenesis and management of perinatal depression.

## 2. Synaptic Plasticity in Pregnancy

It is unclear if synaptic plasticity-related changes are unique in humans or share characteristics with other mammals [4]. Human brains undergo decreases in the overall brain volume during pregnancy with a return to the preconception size during the postpartum period by 6 months [5]. However, recent research findings indicate that there is a symmetrical reduction in extensive grey matter volume in the anterior and posterior cortical midline as well as a few particular sections of the bilateral lateral prefrontal and temporal cortex during pregnancy, lasting for at least 2 years postpartum [6]. Even the pituitary has been noted to increase in size during pregnancy with a return to pre-pregnancy size within the first week of postpartum period [7]. Hormones are thought to be responsible for the morphometric changes during pregnancy. However other factors like genetic expressions, exposure to stress, type of delivery, and feeding methods also influence the structural changes [8]. Another mechanism through which structural plasticity occurs is via the HPA axis (hypothalamic–pituitary axis). Chronic gestational stress can cause dysregulation in the HPA axis, releasing high levels of stress hormones which can in turn regulate synaptic plasticity in the brain, specifically in areas related to the medial prefrontal cortex, nucleus accumbens shell, and basolateral amygdala [9]. Reduced neuroplasticity and the decreased survival of new neurons in the hippocampal area in pregnancy due to adrenal steroids and hormonal fluctuations have been demonstrated in animal studies [10]. Unlike the hippocampus, the sub-ventricular zone has been noted to have increased neurogenesis during pregnancy in rodent studies [11].

In the post-partum period, structural growth has been reported in the areas related to motivation and reward processing (amygdala, hypothalamus, striatum) when assessed in the period between one month postpartum and three to four months postpartum. Other areas that showed structural growth included areas related to the processing of sensory information, empathy, and emotion regulation [12]. These areas include a circuit connecting the amygdala, thalamus, and the orbital and medial prefrontal cortex. Changes in these areas are helpful for the new mothers to adapt to the new demands and responsibilities of motherhood for optimal care of the infant [8].

## 3. Synaptic Plasticity in Perinatal Depression

As there are a lot of changes in the pregnancy and postpartum period in neuroplasticity as well as hormonal fluctuations and stress, it is a vulnerable period for developing depression [13]. Hormonal fluctuations especially in levels of estrogen and progesterone have been implicated in the pathogenesis of postpartum depression. Estrogen is known to enhance serotonergic transmission by increasing the synthesis of serotonin and/or reducing serotonin reuptake. However, in pregnancy, there is a spike in estrogen levels with a sudden and significant decline following delivery, making people susceptible to the development of depression. Other hormonal changes that have a link to post-partum depression include increases in levels of progesterone, cortisol, thyroxine, and thyroid-stimulating hormone, with decreases in the levels of prolactin. However, a causal link has not been established [14]. Alterations in synaptic plasticity in perinatal depression have also been demonstrated in animal studies. An overview of the findings will be described below.

Animal studies usually employ one of the following techniques to simulate perinatal depression: sex hormone withdrawal, induction of gestational stress, and postpartum manipulation [10,15]. Tests used to demonstrate depressive/anxiety states included tests like the forced swim test, sucrose preference test, tail suspension test, open field test, zero maze, light–dark box test, and maternal behavior (nesting or building of the nest, licking, grooming the young, arched-back nursing, lactation, and aggression).

Studies employing sex hormone withdrawal paradigms causing withdrawal of the ovarian sex hormones following a hormone-simulated pregnancy have shown depressive and/or anxiety states along with a reduction in hippocampal cell proliferation and brain-derived neurotrophic factor in female rodents [16]. However, if there was no withdrawal of hormone or if agents like estrogen receptor β agonist or imipramine were given, these changes were not seen [17].

Studies employing exposure to gestational stress in rodents have shown different reactions compared to usual non-gestational chronic stress paradigms. In chronic stress paradigms, a reduction in hippocampal neurogenesis, expression of proteins related to synaptic plasticity, alteration in the density of grey matter, reduction in dendrite length and spine density as well as modified expression of synaptic SNARE (Soluble N-ethylmaleimide-sensitive factor attachment protein receptor) proteins in the hippocampus have been demonstrated [18]. However, gestational stress paradigms have not demonstrated a reduction in hippocampal neurogenesis. In fact, an increase in hippocampal cell proliferation in mid-gestation has been noted [19]. However, reduced frontal cortex integrity has been demonstrated as a result of gestational stress, indicating that the frontal cortex is more vulnerable than the hippocampus to gestational stress [20]. This also implies that women are prone to developing symptoms of depression, anxiety, and cognitive and caregiving difficulties due to a reduction in frontal cortex integrity.

Studies employing postpartum manipulations employed techniques like maternal separation, limited bedding, and the CORT(corticosterone) technique. The maternal separation paradigm involves the separation of the dams(mothers) from their offspring usually from PD (postpartum day) 2 to PD14 or longer. The number of hours differs across studies but usually lasts for 180–360 min [21]. Limited bedding and nesting techniques involve altering the cage environment in the first postnatal week (mostly day 2) by placing the dams and offspring in plastic-coated aluminum mesh bottom 2 cm above the cage floor. The limited bedding provided is usually a thin paper towel which is used by the dam to prepare a rudimentary nest. The cages are usually kept in rooms with good laminar airflow, the droppings are usually dropped to the floor, and the meshes are kept clean by the dams [22]. In the first two techniques, both the offspring and the dams are subjected to stress and the dams would develop depressive-like behavior in the mid- (PD16) and late-postpartum (PD22–26) periods. In terms of synaptic plasticity, a reduction in hippocampal cell proliferation was noted in female rodents who underwent maternal separation daily for 6 h from PD1 to PD14 [23]. The CORT technique involves the administration of daily corticosterone injections to maternal rodents during the entire postpartum duration, which would lead to increased CORT levels in the dams, which in turn would mimic the increased levels of cortisol seen in human mothers with peripartum depression [24]. Studies employing this technique also noted a reduction in hippocampal neurogenesis [25,26]. However, an interesting finding was also noted in the form of the induction of passive coping only when it was given in the postpartum period and not when it was given only or also during the pregnancy stages [26]. This suggests that high levels of glucocorticoids during the postpartum period are more damaging to dams in comparison to high levels of glucocorticoids during gestation. This could be due to different mechanisms of attenuation of the HPA axis during gestation and the postpartum period, making the dams more vulnerable in the postpartum period to the harmful effects of high levels of glucocorticoids.

The GABAergic theory of depression suggests that dysfunction in GABA neurotransmission leads to the development of depression. GABA deficits are specifically more significant in melancholic and treatment-resistant types of depression. Animal models of depression have demonstrated the effect of stress on inducing impairment in GABA neurotransmission. These deficits also cause hyperactivity of the HPA axis and also have a role in impaired neurogenesis in the hippocampus and dentate gyrus. Increased rates of depression in women and vulnerability during the postpartum period are also explained by the dynamic regulation of the GABAergic transmission by pregnancy-related changes in steroid hormone synthesis as well as altered expression of extra-synaptic GABA type A receptors [27].

## 4. Treatment Effects on Synaptic Plasticity

### 4.1. Anti-Depressants

Selective Serotonin Reuptake inhibitors (SSRI) and Serotonin and Norepinephrine Reuptake inhibitors (SNRI) are usually used in the treatment of perinatal depression [28,29]. Chronic stress has a close relationship with the serotonergic system as it causes up-regulation of the serotonin-reuptake transporter (SERT) in the dorsal raphe nucleus and other brain regions receiving serotonergic innervations via the release of CORT. The increased SERT levels cause synaptic deficiency of serotonin, which in turn leads to the development of depressive symptoms [30]. Synaptic deficiency of serotonin also causes neuronal remodeling, causing morphological modifications in the brain. SSRIs reinstate the synaptic availability of serotonin, restoring stress-induced modifications [9]. It is postulated that SSRIs cause anti-depressant activity via restoration of the impaired synaptic plasticity caused by stress and depression [31]. Among the SSRIs, the studies employing citalopram and fluoxetine have found some reversible changes in the synaptic plasticity-related changes developed due to gestational stress. These studies have been highlighted in Table 1. As the doses used in rats and mice differ from humans, the human equivalent dose in mg/kg can be calculated by multiplying the animal dose (rat dose) by 0.162 and the mouse dose by 0.081, as per FDA draft guidelines to account for interspecies scaling [32]. SSRIs are also known to increase BDNF, which explains their role in synaptic plasticity [33]. Chronic stress-induced dysregulation in the HPA axis causes depression and impairment of synaptic plasticity in the brain, which is also believed to be reversed by SSRIs due to their reinstatement of normal HPA axis activity [34]. There is also some evidence that prenatal exposure to SSRIs can increase the risk of development of neurodevelopmental disorders in the offspring. It is hypothesized that the reason for this increased risk is the changes in the hippocampal development induced by SSRIs, as the hippocampus receives significant serotonergic innervations and also plays a key role in cognition and behavior. Maternal stress also contributes to the difference in synaptic plasticity in the offspring. SNRIs also have a similar role to play in the reversal of gestational stress-induced reduction of hippocampal neurogenesis. Overall, reports about the effects of antidepressants on synaptic plasticity are inconclusive and require further studies.

### 4.2. Newer Agents for Perinatal Depression

Emerging evidence suggests that allopregnanolone (a metabolite of Progesterone) has antidepressant properties in postpartum depression [43]. Neurosteroids (including allopregnanolone) undergo a lot of fluctuations throughout pregnancy and the postpartum period. Alterations in levels of allopregnanolone in the postpartum period have been linked to postpartum depression. This implication is further supported by the GABA hypothesis of postpartum depression as well as a positive correlation between the postpartum allopregnanolone levels and modified functional connectivity in the default mode network in the brains of patients with postpartum depression [27,44]. Synthetic neurosteroids for the management of perinatal depression include brexanolone and zuranolone, both of which have potent GABAAR activity. During pregnancy, there is a change in the expression of GABAAR subunits due to increased levels of allopregnanolone, contributing to tonic inhibition. In the postpartum period, there is a sudden drop in the levels of Allopregnanolone, causing an imbalance in the excitation and inhibition ratio, leading to a hyperexcitable state, and causing depression and anxiety. Brexanolone and zuranolone can correct these changes due to their GABAAR activity [43,45].

Other newer agents investigated in perinatal depression include S Ketamine, timosaponin, and resveratrol. Intranasal esketamine (or S Ketamine) is the S enantiomer of ketamine and has antidepressant activity. Emerging evidence indicates that esketamine can cause rapid induction of synaptogenesis and reverse the synaptic deficits produced by depression. It can promote synaptic plasticity in the hippocampus [46]. It has also been found to cause rapid release of brain-derived neurotrophic factor (BDNF), contributing to its quick antidepressant action [47].

Timosaponin B-III is a saponin isolated from the anemarrhenae rhizome. A mouse model of postpartum depression showed that timosaponin has antidepressant activity via regulation of the BDNF signaling pathway, regulation of inflammatory cytokines, and the regulation of synaptic plasticity-related proteins [48].

Resveratrol is an activator of Silent Information Regulator 1 (Sirt1), which is known to contribute to the pathogenesis of depression. It has been shown to reduce both lipooligosaccharide-related depressive behavior as well as chronic glucocorticoid exposure-related depressive behavior via enhanced hippocampal neurogenesis [49,50].

### 4.3. Neuromodulation and Synaptic Plasticity

Non-invasive neuromodulation or non-invasive brain stimulation (NIBS) therapies include the administration of transcranial electric stimuli or magnetic stimulation to modulate brain activity at cortical and subcortical levels [51,52]. The common NIBS used in the treatment of depression include therapies like electroconvulsive therapy (ECT), transcranial magnetic stimulation (TMS), and transcranial direct current stimulation (TDCS). These methods are safe and well-tolerated in pregnancy and postpartum period, and are commonly utilized in this population.

#### 4.3.1. Electroconvulsive Therapy (ECT)

ECT involves the administration of electrical stimuli to induce a therapeutic tonic-clonic convulsive seizure. It has well-established effectiveness in the treatment of severe depression, catatonia, high suicide risk, and treatment-resistant illnesses including depression [53]. It is a safe and effective treatment option for perinatal depression especially when rapid alleviation of symptoms is desired, and when there is resistance to pharmacological treatment [54].

Animal studies have consistently demonstrated the ability of ECT to enhance hippocampal neurogenesis and resultant antidepressant actions in both rodent and non-human primates [55,56]. Other synaptic plasticity-related changes like synaptogenesis, gliogenesis, and angiogenesis have been reported too [57]. A meta-analysis suggested the effects of ECT on enhancing the hippocampal volumes in human brains and highlighted its age-dependent effects, implying that the changes were more prominent in younger individuals [58]. A human study has shown the effects of ECT on synaptic plastic changes in the dentate gyrus, mediating a role in short-term antidepressant action [59]. However, there is a dearth of such studies in the perinatal population.

#### 4.3.2. Transcranial Magnetic Stimulation(TMS)/Repetitive Transcranial Magnetic Stimulation (rTMS)

rTMS involves the application of alternating magnetic fields to stimulate neurons in the brain. High-frequency rTMS (stimulation at frequency > 5 Hz) increases cortical excitability, whereas low-frequency rTMS (stimulation at frequency < 1 Hz) decreases neuronal excitability [60]. In depression, the dorsolateral prefrontal cortex (DLPFC) has been found to be hypoactive. High-frequency rTMS over the left DLPFC is found to be effective in depression. Other areas tried include low-frequency rTMS stimulation over the right DLPFC, low-frequency rTMS over the right parietal cortex, and high-frequency rTMS over the cerebellum etc. However, the results are not as robust as rTMS over the left DLPFC in patients with depression. rTMS has been widely used for the treatment of depression and is also a safe and effective treatment option in the perinatal period [61].

The long-term effects of rTMS are postulated to be due to the induction of cortical plasticity as a result of the induction of genes and the synthesis of proteins. Animal studies have demonstrated that rTMS induces long-lasting functional as well as structural changes in excitatory postsynapses, as well as specific inhibitory postsynapses on some principal neurons [62,63,64].

#### 4.3.3. Transcranial Direct Current Stimulation (tDCS)

tDCS involves the application of low-amplitude current (1–2 mA) through bio-conducting electrodes (cathode and anode) placed on the scalp. Anodal tDCS causes cortical excitability by inducing neuronal depolarization, whereas cathodal tDCS reduces cortical excitability by inducing neuronal hyperpolarization [65]. In the treatment of depression, the anode is usually placed over the left dorsolateral prefrontal cortex (dlPFC), and the cathode is placed over the contralateral supraorbital area, corresponding to F3 and FP2 as per the international 10–20 EEG system [66]. It is effective and safe in the management of depression and has been administered in the perinatal population without significant adverse effects. However, large-scale studies are needed to establish its efficacy in the perinatal population [67].

Studies have shown the effects of tDCS on strengthening synaptic connections through a mechanism similar to long-term potentiation that lasts longer than the period of stimulation. Its effects on the augmentation of synaptic plasticity through inducing the secretion of BDNF have been reported [68,69].

### 4.4. Non-Pharmacological Interventions for Depression

Non-pharmacological interventions for depression include psychotherapy, physical exercise, yoga, and bright-light therapy. All these interventions are effective and safe in the postpartum period too.

#### 4.4.1. Psychotherapy

Psychotherapy in the form of behavioral activation, cognitive behavior therapy, supportive therapy, interpersonal therapy, mindfulness-based therapy, and acceptance and commitment therapy are effective in depression. Some of the common techniques include ventilation, catharsis, cognitive restructuring, reality testing, and new learning etc. The environment produced during psychotherapy helps in nurturing the brain of the patient to grasp and learn new techniques. Positive therapeutic relationship, empathy, emotional regulation, and stress modification also involves neuroplastic changes. Neuronal growth, enhancement of neuronal connectivity, and neurogenesis are considered the essential mechanisms of all forms of learning and change. Neurogenesis occurs in different parts of the brain, such as the hippocampus, amygdala, and temporal and frontal lobes during the learning process in psychotherapy [70]. Psychotherapy has also been reported to affect the cerebral metabolic rate (for example, increased pre-frontal metabolism and decreased activation of the limbic system), improve the metabolism of serotonin, affect the thyroid axis, cause gene modifications, improve neuronal integrity, and cause modifications in dendritic lengths, spine densities and glial activities [71]. All these changes are long-lasting and help in the long-term modification of emotions and behaviors.

#### 4.4.2. Physical Exercise

Epidemiological studies have demonstrated a significant link between exercise (aerobic exercise, mind-body exercise and resistance training, etc.) and reduced rates of depression. Physical exercise has been found to increase the levels of BDNF, TrkB (tyrosine receptor kinase B) receptor, VEGF (Vascular endothelial growth factor), and IGF-1 (insulin-like growth factor 1) levels in the hippocampal dentate gyrus, and it has been shown to improve learning and memory. It has also been noted to modify brain structure, stimulate certain brain areas, induce neurogenesis, and modify behavior [72]. As the hippocampus is also associated with depression-like changes, exercise should ideally help improve symptoms of depression. However, there are mixed results regarding the same, and the effects of exercise on synaptic plasticity in depression are inconclusive and warrant further research [73].

#### 4.4.3. Yoga

Yoga is an alternative form of treatment that includes components of yoga asanas, pranayamas, and meditation, and it has shown benefits in depression, anxiety, and stress reduction. It has a role in mind–body integration and healing, as it has also been shown to increase the levels of BDNF and decrease the inflammatory markers andcomplement components such as C1q (known to have a significant role in synaptic pruning), Factor H, and properdin seen in depression [74,75]. It has also been shown to improve the homeostasis of serotonin, modify gene expressions, modify HPA axis changes, and increase neuroplasticity. Long-term practice of yoga has also been shown to induce changes in the grey matter thickness and duration of the leukocyte telomere, and to contribute to a reduction in stress-related and inflammatory conditions including depression [76].

#### 4.4.4. Bright-Light Therapy (BLT)

Bright-light therapy usually involves treatment starting with 10,000 lux for 30 min every morning, but the duration and intensity may be modified. Bright-light therapy is a good option for treatment of perinatal depression (especially the winter depression type) because of its low cost, home-based form, and much lower side effect profile compared to pharmacotherapy. Additional benefits include its ability to alleviate fatigue and insomnia, modulate in estrogen levels, regulate serotonergic metabolism, and resynchronize the circadian system [77]. Early morning daily BLT for 4 weeks has been shown to cause modifications in the expression of neuroinflammatory markers such as CD11b, tumor necrosis factor -α (TNF-α), and interleukin 6(IL6), and neuroplasticity markers (BDNF and TrkB) in the cortico-limbic brain regions (mPFC, BLA, and hippocampus) [78].

## 5. Conclusions

Synaptic plasticity is disrupted in perinatal depression due to a combination of various factors like stress, dysregulation of the HPA axis, and hormonal fluctuations. The role of antidepressants in reversing changes in synaptic plasticity is still unclear, and warrants further research. Non-pharmacological interventions such as psychotherapy, physical exercise, yoga, and bright-light therapy have shown promising effects on synaptic plasticity, and are relatively safer methods of treatment. Newer methods of treatment like neuro-steroids and non-invasive neuromodulation appear to have anti-depressant properties through their role in enhancing synaptic plasticity, and need to be explored further, especially in the perinatal population.

## Figures and Tables

**Table 1:** Animal studies of the effects of anti-depressants on synaptic plasticity.

Study	Drug	Method	Results
Haim et al., 2016 [9]	Citalopram	Pregnant rats exposed to gestational stress paradigm. Postpartum rats were randomly assigned to receive citalopram 10 mg/kg (HED = 97.2 mg) or saline. A forced swim test was carried out (FST), following which Golgi staining (post 24 h) and microscopic analysis of brains were performed.	Citalopram reduced depressive-like behavior and also reversed gestational stress-induced structural plasticity-related changes in the postpartum nucleus accumbens shell and mPFC, but not in the BLA.
Salari et al., 2016 [35]	Fluoxetine	Gestationally stressed and non-stressed mouse dams were treated with oral fluoxetine 5 mg/kg (HED = 38.88 mg) from gestational day 10 to lactation day 20. Sucrose preference, a forced swim test, zero maze, and light–dark box tests were employed. Stress-induced corticosterone levels were collected.	Depressive-like behavior and HPA overactivity were reversed by fluoxetine.
Gemmel et al., 2016 [36]	Fluoxetine	Gestationally stressed and non-stressed rat dams were treated with fluoxetine 5 mg/kg (HED = 48.6 mg). At weaning, the maternal brains were analyzed.	Fluoxetine reduced methylation as well as serotonergic functioning in the maternal hippocampus. Fluoxetine reversed the effect of stress by restoring neuronal activity and serotonergic functioning in the PFC of the maternal brain.
Pawluski et al., 2012 [37]	Fluoxetine	Gestationally stressed and non-stressed rat dams were treated with fluoxetine 5 mg/kg (HED = 48.6 mg) or vehicle for 28 days (PD1 to PD28). The maternal care, anxiety-like behavior, depressive-like behavior, corticosterone levels, corticosterone binding capacity and histology of maternal rat brains (after PD28) were assessed.	Fluoxetine reduced the morning levels of corticosterone and CBG in dams and increased hippocampal neurogenesis in gestationally stressed dams.
Workman et al., 2016 [38]	Fluoxetine	Nulliparous and postpartum female rats were divided into four groups that received 21 d of injections of CORT or oil plus FLX 10 mg/kg (HED = 97.2 mg) or saline. FST, radioimmunoassay, maternal behavior assessment, immunohistochemistry, and microscopic analysis of brains were carried out.	Fluoxetine increased neurogenesis in the ventral hippocampus in the nulliparous rat group but not in the postpartum dam group.
Gobinath et al., 2018 [25]	Fluoxetine	Corticosterone-stressed postpartum female rats were assigned to four groups: FLX 10 mg/kg (HED = 97.2 mg) + exercise, Only FLX, Saline + Exercise, and Only Saline. FST, radioimmunoassay, maternal behavior assessment, immunohistochemistry, serum CORT assay, and microscopic analysis of brains were carried out.	Fluoxetine reduced hippocampal neurogenesis. Only exercise and a combination of FLX + exercise increased neurogenesis in postpartum dams.
Gemmel et al., 2018 [39]	Fluoxetine	Female rats were put under chronic unpredictable stress paradigms for 3 weeks before breeding. They were later given oral fluoxetine 10 mg/kg (HED = 97.2 mg) or vehicle. Maternal behavior, serum cortisone, serum CBG, and histology of dam brains were carried out.	Fluoxetine significantly increased the number of immature neurons in the dorsal hippocampus of the dams, more in non-gestationally stressed dams.
Kott et al., 2018 [40]	Sertraline	Gestationally stressed (CORT paradigm) dams were randomly assigned to receive sertraline 20 mg/kg (HED = 194.4 mg) or vehicle for either up to GD16 or till parturition. CORT assay, FST, OFT, maternal care observation, immunohistochemistry, and microscopy were carried out.	Sertraline had no impact on hippocampal neurogenesis in the dams.
Pawluski et al., 2020 [41]	Sertraline	Pregnant and non-pregnant female rats were given sertraline 2.5 mg/kg/day (HED = 24.3 mg) or 10 mg/kg/day (HED 97.2 mg) or vehicle for the last half of pregnancy (10 days). Immunohistochemistry was performed.	There was a negative association between the serum sertraline levels and the measures of hippocampal neurogenesis (cell proliferation and immature neurons), which were specifically pronounced in non-pregnant females.
Belovicova et al., 2017 [42]	Venlafaxine	Gestationally stressed rats were treated with oral venlafaxine 5 mg/kg(HED = 48.6 mg) twice a day. Maternal behavior was evaluated within 5 min observations twice a day and again after 8 weeks in a single 15 min session. Immunohistochemistry and microscopy were performed.	Venlafaxine reversed the reduction in hippocampal neurogenesis induced by gestational stress in dams.

mPFC = medial pre-frontal cortex’; BLA = basolateral amygdala; FST = forced swim test; OFT = open field test, CORT = corticosterone; GD = gestational day; FLX = fluoxetine; PD = postpartum day; HPA = hypothalamic–pituitary–adrenal; HED = human equivalent dose in mg/day with reference weight of 60 kg.

## Data Availability

No new data were created or analyzed in this study. Data sharing is not applicable to this article.
